# Developmental role of acetylcholinesterase in impulse control in zebrafish

**DOI:** 10.3389/fnbeh.2015.00271

**Published:** 2015-10-16

**Authors:** Matthew O. Parker, Alistair J. Brock, Ari Sudwarts, Muy-Teck Teh, Fraser J. Combe, Caroline H. Brennan

**Affiliations:** ^1^School of Biological and Chemical Sciences, Queen Mary University of LondonLondon, UK; ^2^School of Health Sciences and Social Work, University of PortsmouthPortsmouth, UK; ^3^Centre for Clinical and Diagnostic Oral Sciences, Institute of Dentistry, Barts and The London School of Medicine and Dentistry, Queen Mary University of LondonLondon, UK

**Keywords:** 5-choice serial reaction time, impulsivity, acetylcholinesterase, Dopamine D2 receptor, zebrafish

## Abstract

Cellular and molecular processes that mediate individual variability in impulsivity, a key behavioral component of many neuropsychiatric disorders, are poorly understood. Zebrafish heterozygous for a nonsense mutation in *ache* (ache^*sb55/*+^) showed lower levels of impulsivity in a 5-choice serial reaction time task (5-CSRTT) than wild type and ache^+∕+^. Assessment of expression of cholinergic (nAChR), serotonergic (5-HT), and dopamine (DR) receptor mRNA in both adult and larval (9 dpf) ache^*sb55/*+^ revealed significant downregulation of *chrna2, chrna5*, and *drd2* mRNA in ache^*sb55/*+^ larvae, but no differences in adults. Acute exposure to cholinergic agonist/antagonists had no effect on impulsivity, supporting the hypothesis that behavioral effects observed in adults were due to lasting impact of developmental alterations in cholinergic and dopaminergic signaling. This shows the cross-species role of cholinergic signaling during brain development in impulsivity, and suggests zebrafish may be a useful model for the role of cholinergic pathways as a target for therapeutic advances in addiction medicine.

## Introduction

The identification of endophenotypes, as quantifiable, core components of complex behavioral traits and disease phenotypes makes genetic analysis of the pathogenesis of neuropsychiatric disease more tractable in both humans and model organisms (Burmeister et al., 2008). One such potential endophenotype is impulsivity (Urcelay and Dalley, 2012). Impulsivity not only is the hallmark symptom of a number of neuropsychiatric disorders (ADHD, addiction) but, in the case of addiction, has been shown to predict patterns of relapse and compulsive drug seeking in rats (Belin et al., 2008).

Despite the well-established role in a number of neuropsychiatric disorders, the cellular, and molecular mechanisms that underlie impulsivity are not well-understood. The cholinergic system, in particular cholinergic projections from the PFC, has long been implicated in sustained attention (Sarter et al., 2001). For example, IgG-saporin lesions of cholinergic neurons in the basal forebrain reduce sustained attention (Mcgaughy and Sarter, 1998), while systemic administration of the nAChR agonist nicotine improves performance accuracy and reduces omissions on the 5-CSRTT (Blondel et al., 2000; Hahn and Stolerman, 2002; Young et al., 2004). In addition, infusions of scopolamine (mAchR antagonist) into the medial pre-frontal cortex (mPFC), and systemic mecamylamine (nAchR antagonist) reduce response accuracy (Robbins et al., 1998). The effects of chronic elevation of ACh, however, are less clear, although Grottick and Higgins (2000) found that improved performance accuracy is apparent with chronic nicotine exposure. The effects of genetic alteration of ACh activity have not previously been tested, particularly with respect to premature responding on the 5-CSRTT.

Notwithstanding their small size, low housing costs and prolific breeding, there now exists a number of genetic tools for zebrafish research, including N-ethyl-N-nitrosourea (ENU) mutagenized lines, extensive sperm libraries, and a number of GFP/RFP lines. Despite anatomical differences between the fish and their mammalian counterparts, key neurochemical pathways are well-conserved between the species (Guo, 2004); for example, the ascending and descending midbrain catecholeminergic pathways (Guo et al., 1999).

Here, we tested the performance of *ache* (the gene that codes for acetylcholinesterase; AChE) deficient (ache^*sb55/*+^) zebrafish, for performance characteristics on the 5-CSRTT, a task designed to test aspects of impulse control through examination of anticipatory responding. ache^*sb55*^ contain a point mutation close to the catalytic site of the enzyme resulting in a replacement of serine 226 by an asparagine. Serine 226 is conserved in all *ache* gene family members, and is important for catalytic activity (Behra et al., 2002). Chronic alterations in cholinergic signaling with the AChE inhibitor chlorpyrifos has previously been demonstrated to increase impulsivity, make cholinergic signaling an interesting target for inquiry into the molecular mechanisms underlying impulse control (Middlemore-Risher et al., 2010; Cardona et al., 2011; Oca et al., 2012). Zebrafish have previously been shown to respond well on the 5-CSRTT (Parker et al., 2012, 2013, 2014).

## Materials and methods

### Ethics statement

All experimental procedures, including drug dosing, and behavioral testing, were carried out under the Animals (Scientific procedures) Act (1984). The procedures carried out conformed both to local ethical guidelines and to the terms of a project license from the UK Home Office. In addition, all experiments were approved by the Queen Mary Animals Welfare and Ethical Review Board.

### Subjects

Twenty-nine (*n* = 10 ache^*sb55/*+^ (Ninkovic et al., 2006), *n* = 19 Tubingen wild-type), adult zebrafish (age = 6 months; mixed sex) were selected for the first part of the study (5-s fixed interval PSI), and 12 adult zebrafish (age = 5 months; mixed sex; *n* = 5 ache^*sb55/*+^; *n* = 7 ache^+∕+^) were selected for the second part (Variable PSI). All were sourced initially from the Sanger Institute (Cambridge, UK), and bred and reared in the aquarium facility at Queen Mary University of London according to standard protocols (Westerfield, 1993). During the entire experimental period, fish were fed artemia/bloodworm mix during testing trials, and this was supplemented with flake food/artemia in the evenings and at weekends.

### Apparatus

**Figure 1:**
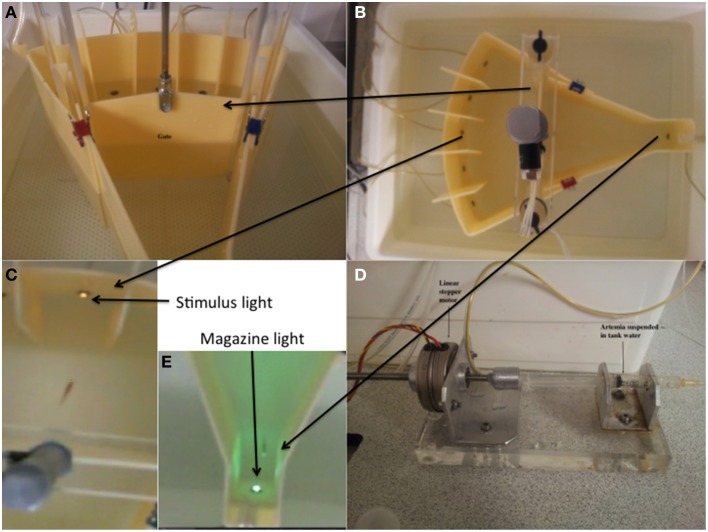
**Five-choice serial reaction time task testing unit and the constituent parts. (A)** The pneumatic gate mechanism. **(B)** The 5-choice apparatus viewed from the perspective of the camera. **(C)** The stimulus light area. The stimuli were 5 white LEDs. **(D)** Food was delivered via activation of a linear stepper motor driving the plunger of a 1.5 ml plastic syringe. The food (liquidized bloodworm and brine-shrimp) was delivered to the fish through 1 mm latex catheter tubing. **(E)** The food delivery area and magazine. This comprised a green LED to act as a stimulus to signal food availability. Adapted from Parker et al. (2013).

Figure 1 displays the 5-CSRTT tanks used in the study. The shell of the testing tanks was constructed from opaque acrylic, as were the central gates. The lights were LEDs (magazine light green, stimulus aperture lights yellow). The reinforcer used was artemia liquidized with bloodworm, suspended in aquarium-treated water (R-O water with added salts). The food was delivered via a plastic syringe fitted with a 1 mm diameter rubber catheter tube, which was driven by a linear stepper motor (Figure 1).

### General procedure

The main procedure is an extension and modification of the commonly used rodent 5-CSRTT, and has been described in detail elsewhere (Parker et al., 2012, 2013, 2014).

### Pre-training

Prior to commencing training, all subjects were habituated to the test room for 1 week to acclimate to the conditions. All pre-training, training and testing was carried out Monday–Friday (0800–1800), with the exception of the final stage (Stage 8, see Table 1), which was also carried out Saturday and Sunday. Training was divided into eight distinct stages (see Table 1).

**Table 1:** **Procedure for pre-training and training during 5-CSRTT**.

**Stage**	**Procedure**	**Description**	**Timecourse**
Pretraining	1. Acclimation	All apparatus lights on, barrier raised	Days 1–5
	2. Magazine training	Barrier down. Magazine light on 30-s. Food available on entry to magazine. 10-s ITI.	Days 6–10
	3. Response aperture orientation	All stimulus lights illuminated. Barrier lifted, all stimulus lights illuminated. Entry to any hole reinforced with illumination of magazine light. Food delivered on entry to magazine. Barrier down after correct response. 10-s ITI (stimulus lights off, barrier down)	Days 11–15
5 CSRTT	4.30-s stimulus training	Trial commences with barrier lifted, followed by 1-s pause (ITI). Stimulus lights illuminated in random order (30-s), followed by 1-s limited hold period (stimulus light off). Responses during the stimulus or the limited hold conditionally reinforced with illumination of magazine light. Food delivered on entry to magazine. Barrier down after correct response. Ten second pause following magazine entry (stimulus lights off, barrier down). Subsequent trial initiated following next magazine entry following this pause	Days 16–35
	5.10-s stimulus training	As above (4), but stimulus light illuminated for 10-s	Days 36–45
	6.5-s stimulus light, 2-s ITI	As above (4), but stimulus light illuminated for 5-s, and ITI increased to 2-s	Day 46–55
	7.5-s stimulus light, 5-s ITI (Baseline)	As above (6), but ITI increased to 5-s	Day 56–60
Testing	8. Long ITI training	Day 1–Long ITI (as above (7; baseline), but ITI increased to 7-s). Days 2–3–Baseline (as above (7). Day 4–Long ITI, Days 5-6–Baseline. Day 7–Long ITI	Day 61–68

During stages 1–3 (pre-training) data were collected and examined to ensure that all animals were receiving food during training. Any that did not perform the task (e.g., froze in the tank or did not approach the lights; *n* < 2 on any given session) had their food supplemented immediately after the session. During acclimation (Stage 1), fish were placed individually into the test tanks for 30-mins. During this all lights were illuminated and the gate was open. Immediately after acclimation, the fish were trained to enter the food magazine (Stage 2). During this stage, the gate remained closed at all times. The magazine light was illuminated for 30-s intervals, during which entry to the magazine resulted in the light turning off, and a small delivery (~20 μl) of artemia/bloodworm mix. In Stage 3 the fish were trained to approach the response apertures. Here, the gate opened to reveal all of the response apertures illuminated, and entry to any one of the apertures was conditionally reinforced with illumination of the magazine light. Subsequent entry to the food magazine was reinforced with artemia/bloodworm mix. During Stage 3 (response aperture orientation) only fish that completed 20 or more correct trials were taken forward to 5-CSRTT training.

### Five-choice serial reaction time task: phase 1

After a 2-min habituation period, the magazine light was illuminated, and entry to the food magazine initiated the trial sequence after an inter-trial interval (ITI) of 20-s^1^. This ITI always followed food delivery, and allowed the fish time to consume the reinforcer ration. After 20-s, the gate was raised, and one of the stimulus apertures was illuminated after a pre-stimulus interval (PSI). Entry to the correct aperture during the stimulus illumination, or during a brief pause thereafter (limited hold; LH), were conditionally reinforced by illumination of the magazine light, and the trial ended when the fish collected the food. All training sessions lasted 30-mins. For the first 4 weeks (Stage 4) the fish were trained with 30-s stimulus duration, a PSI of 1-s and a 1-s limited hold period. At all times during training and testing, the magazine light remained illuminated for 30-s following a correct response, after which magazine entry was not reinforced. During the second stage of 5-CSRTT training (Stage 5) the stimulus duration was reduced to 10-s, the PSI was increased to 5-s and limited hold remained at 1-s. The criterion for moving from each stage to the next was that the fish had reached a steady-state response, operationalized as completing >20 trials per session over 5-consecutive sessions. Any fish not meeting this criterion were excluded from the subsequent stage.

### Long PSI stage

There were three long PSI sessions, during which the PSI was increased to 7-s. All other test parameters remained the same as during Stage 5 (stimulus duration = 10-s, limited hold = 1-s). The three long PSI sessions were interspersed by two baseline sessions (Stage 5; PSI = 5-s, stimulus duration = 10-s, limited hold = 1-s). During the long PSI sessions, the length of the session was increased to 35 min. The criterion for a fish progressing to the long PSI phase of the experiment was that they reached steady state responding, again, operationally defined as having completed five sessions of >20 trials prior to testing. Any fish that did not meet this criterion were excluded from the testing phase.

### Five-choice serial reaction time task: phase 2

For the second phase of the experiment, we trained a group of experimentally naïve fish (*n* = 5 ache^*sb55/*+^; *n* = 7 ache^+∕+^) in an identical manner to that described above for stages 1–4. For Stage 5, we introduced 5-s variable interval (VI) PSI. All other timings were the same as in Phase 1, Stage 5 (stimulus duration = 10-s, limited hold = 1-s). There was no Long-PSI stage in Phase 2.

### Acute exposure to AChe antagonist, and nAChR and mAChR agonists

Trained fish (wild-type from Phase 1) were selected for the drug administration phase. The exposure schedule was organized according to a full crossover design, with each fish receiving each of the drugs over a 1-week period. Fish were initially re-trained (2-weeks) in the absence of drug to establish steady-state baseline performance (>20 reinforced trials/session, for 5 sessions). The 5-CSRTT was as before in Stage 5 (see above: stimulus-duration = 10-s, PSI = 5-s, LH = 1-s), except that in this phase we employed a variable interval (VI) 5-s PSI. During the first experiment, there was no difference between the strains during the long PSI trials, but there was a difference during the earlier stages of training. Therefore, we chose to increase the complexity of the task by using a VI-PSI during the entire training period. Immediately prior to training, fish were immersed in a pre-treatment tank (1 L) either in the drug solution or in aquarium-treated H_2_O for 20-mins. Drugs (nicotine: 1.54 μM [Sigma-Aldrich, UK]; pilocarpine [Sigma-Aldrich, UK]: 8.64 μM; donepezil [Sigma-Aldrich, UK]: 2.63 μM) were dissolved in aquarium-treated H_2_O. Doses of donepezil, nicotine and pilocarpine were selected based on previous work on attention/impulsivity (Day et al., 2007; Brembs, 2009; Cardona et al., 2011). The dose of donepezil was also based on an initial assessment of brain levels of ACh and AChE following drug administration to determine a dose that best reflected the ACh and AChE levels in ache^*sb55*∕+^ (Ninkovic et al., 2006).

Brain activity of AChE and brain levels of ACh were assessed in wild-type fish exposed to 2.63 μM donepezil or aquarium-treated H_2_O for 20 mins using a fluorescence-based approach (George et al., 1961). Following exposure to drug fish were placed in a recovery tank for 5-min, and then killed by immersion in ice water. Brains were immediately removed, weighed and homogenized in ice-cold Tris-HCl (pH 8). Samples were then centrifuged (20-min at 13,000 rpm) and AChE and ACh was assessed from the resulting supernatant using Amplex Red Acetylcholine/Acetylcholinesterase assay kit (Molecular Probes, Invitrogen Detection Technologies, Paisley, UK) according to manufacturer's instructions. Briefly, AChE converts ACh into choline, which is then oxidized by choline oxidase to betaine and H_2_O_2_. Brain activity of AChE and brain levels of ACh were measured using 10-acetyl-3, 7-dihydroxyphenoxazine, a flourogenic probe for H_2_O_2_. All ACh and AChE samples were examined in duplicate against standards and fluorescence was measured on a fluorescence microplate reader (FLUOstar OPTIMA, BMG LABTECH, Cary, NC). Following exposure to 2.63 μM donepezil, the levels of ACh were found to be higher in the drug group [11.8 nM/g vs. 7.1 nM/g; *t*_(8)_ = 2.81, *P* = 0.02], which was directly comparable to levels seen in the ache^*sb55/*+^ thus validating the dose used (Ninkovic et al., 2006).

The exposure schedule was as follows: Week 1: drug A, Week 2: recovery (no drug), Week 3: drug B, Week 4: recovery, Week 5: drug C. As stated, each fish was tested in the presence of each of the three drugs, the order of which was counterbalanced across weeks.

### Gene expression changes in ache^sb55/+^

We collected embryos from 4 × ache^*sb55*∕+^ in-crosses. All homozygous individuals were removed at 72 hpf (easily identifiable by morphological features and lack of motor activity) leaving petri dishes with ~2/3 heterozygous individuals. We also collected embryos from 4 × ache^+∕+^ in-crosses for comparison. Reference genes used were β-*actin, ef1*α, and *rpl13*α based on previous findings findings (Tang et al., 2007). Target genes used are listed in Table 2. All embryos were manually sorted to ensure all were at the same developmental stage over the first 72 hpf, and grown to 9 dpf in petri dishes (~40/dish) in an incubator (28°C). At 9 dpf embryos were terminally anesthetized in MS-222, and placed in RNAlater until assay (4°C). Eight batches of *n* = 3 embryos per strain (ache^*sb55*∕+^ and ache^+∕+^) were lysed in 200 μl Lysis buffer with 2 μl Proteinase K for 30–45 min (55°C). mRNA was isolated using 40 μl Dynabeads® Oligo(dT)_25_ according to manufacturer's instructions. Ten adult (6 months) brains (*n* = 5 ache^*sb55/*+^; *n* = 5 ache^+∕+^) were homogenized in 400 μl Lysis buffer with 4 μl Proteinase K for 30-min (55°C). mRNA was isolated using 80 μl Dynabeads® Oligo(dT)_25_ according to manufacturer's instructions. All qPCR reactions were carried out in triplicate. 1 μl of cDNA and 1.5 μl each of forward and reverse primers (see Table 2) were added to 5 μl SYBR® Green PCR Master mix and run in a 384-well plate format (Roche Diagnostics). Method reported in full elsewhere (Gemenetzidis et al., 2010) (Teh et al., 2013).

**Table 2:** **Primer pairs for all reference and target genes examined in quantitative real-time PCR analysis**.

**Primers**	**Gene name**
**REFERENCE GENES**	
*β-actin-F*	CGA GCT GTC TTC CCA TCC A
*β-actin-R*	TCA CCA ACG TAG CTG TCT TTC TG
*rpl13a-F*	TCT GGA GGA CTG TAA GAG GTA TGC
*rpl13a-Ft*	AGA CGC ACA ATC TTG AGA GCA G
*eF1a-F*	CTG GAG GCC AGC TCA AAC AT
*eF1a-R*	ATC AAG AAG AGT AGT ACC GCT AGC ATT AC
**TARGET GENES**	
*adora2aa-F*	CTT GAG CGC AGG AAC CAG AG
*adora2aa-R*	CGC GCA CTG AGA GAT GAC AG
*chma2-F*	GCG GAAAAC CGG ATA AAA ACA CTC
*chrna2-R*	AGT TTG TCC TCT GCG TGT GCA T
*chma3-F*	TGT ACA TCC GCC GAT TAC CGC T
*chma3-R*	TCC GCA GTC GGA GGG CAG TA
*chma4-F*	TTA CAA GAG GTT TGG GCG CT
*chrna4-R*	ACA GAC CAG TAG ATC ATC ACT CC
*chrna5-F*	GGC TCC CAG GTC GAC ATT
*chrna5-R*	AAC CCC GGT TAC CAG TGG CCT
*chrna6-F*	CTT TGG GCC TCT TCC TGC AA
*chrna6-R*	TCA GAG TCT TGA TGT AGT GAC GG
*chrna7-F*	ACC GTG TCA CAT TGT TCA TTC TC
*chma7-R*	ACA GGT CTC TCC AGT GGG TTA
*chrnb2-F*	GGC TGC CTG ATG TTG TTC TT
*chrnb2-R*	TGG TGG CAA CCA GAA GAC ACT T
*chrnb3-F*	CAG GAG TCA ACC TCC GCT TT
*chrnb3-R*	TGA ATC TGA ACG CAC TGG CT
*chrnb4-F*	TGA TCA CAT GAT GGG GAA TGA CG
*chrnb4-R*	CAC CAC ACA CAC GAT CAC AAA G
*drd1-F*	TGG TTC CTT TCT GCA ACC CA
*drd1-R*	AGT GAT GAG TTC GCC CAA CC
*drd2-F*	TCC ACA AAA TCA GGA AAA GCG T
*drd2-R*	CAG CCA ATG TAA ACC GGC AA
*drd3-F*	ATC GAG TTT CGC AGA GCC TT
*drd3-R*	TCC ACA GTG TCT GAA AGC CG
*htr1aa-F*	GGA GCC CGC CAT GCG TCT T
*htr1aa-R*	CGT CGC GTT CCC GCT CCA A
*oprm1-F*	CCG TAT GTG ACA GGA CGC CA
*oprm1-R*	TTT CCC ACC AGT CCC ATC ACA
*slc6a2-F*	AGG TGA CAT TGT TTG AGA TGT CTT
*slc6a2-R*	TGT CTT GGT AGT GTC AAG TTG T
*slc6a3-F*	TAT GTG GTC CTG ACC GTG CT
*slc6a3-R*	CAC ATG TGT AGG CGC AGG AA
*slc6a4-F*	GCC ACA GGC CCC GCT GTT A
*slc6a4-R*	ACC AGG GGC GAA GCC AAG CA

### Data analysis

5-CSRTT data were fitted to general linear models (fit by REML), with time (5-CSRTT phases 1–5) and strain (either ache^*sb55/*+^ vs. ache^+∕+^ or ache^*sb55/*+^ vs. wild-type) as fixed effects. In the drug administration phase, drug (four-levels, nicotine, pilocarpine, donepezil, and control) was added as a fixed factor, with ID and day as random effects. In each case, the dependent measure was calculated from performance in the 5-CSRTT:

- Correct; calculated as: $\frac{correct}{\left(correct\;+\;incorrect\right)}$
- Omissions; calculated as: $\frac{omissions}{\left(correct+incorrect\;+\;omissions\right)}$
- Premature; calculated as: $\frac{premature}{\left(correct\;+\;incorrect\;+\;omissions\;+\;premature\right)}$

*Post-hoc* Tukey tests were carried out to examine main effects and interactions of 5-CSRTT data.

Finally, to test the difference between levels of mRNA expression in larvae and adult ache^*sb55/*+^ and ache^+∕+^ siblings, we carried out a series of Mann–Whitney *U*-tests, with strain (ache^*sb55/*+^ vs. ache^+∕+^) as the independent variable and target gene expression, relative to reference genes, as the dependent variables. For mRNA expression data, *P* values were estimated following Bonferroni correction for multiple comparison. Effect sizes for all differences in expression were also calculated using the Grissom and Kim (2012) method. Descriptive statistics are reported as mean ± SEM unless otherwise stated. A type-1 error rate of α = 0.05 was adopted for all statistical tests. All data were analyzed using IBM SPSS Statistics v.21 for Macintosh.

## Results

### ache^*sb55*/+^ show higher levels of responding during pre-training

**Figure 2:**
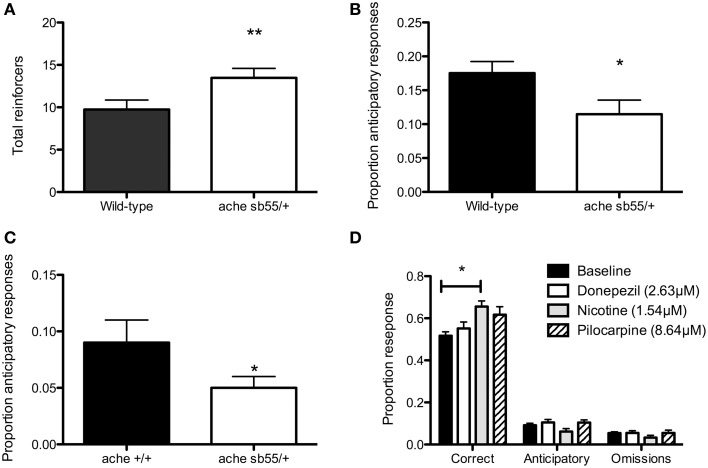
**Five-choice serial reaction time task data. (A)** ache ^*sb55*∕+^ receive more reinforcers in the stimulus-light training session that TU wild-type fish; **(B)** ache^*sb55/*+^ perform a lower proportion of anticipatory responses during 5-CSRTT training than TU wild-type; **(C)** ache ^*sb55/*+^ perform a lower proportion of anticipatory responses in 5-CSRTT than ache ^+∕+^; **(D)** 1.54 uM nicotine increases proportion of correct responses during 5-CSRTT in TU wild-type fish. Note: ^*^P < 0.05; ^**^P < 0.01.

The ache^*sb55/*+^ heterozygotes were selected by systematic in-crosses, the mutation being homozygous-lethal. There was a main effect for day, *F*_(4, 35)_ = 3.42, *P* < 0.02. *Post-hoc* pairwise comparisons revealed that there was a significant increase after Day 1 (*P*s ≤ 0.05), but no change thereafter (*P*s > 0.6). There was also a significant main effect for strain, *F*_(1, 85)_ = 5.61, *P* < 0.01, with the ache^*sb55/*+^ making significantly more response than the wild-type (Figure 2A). There was no day × strain interaction (*F* < 1). Of the original 39 fish, 3 of the ache^*sb55/*+^ (30%) and 8 of the 19 wild-type (42%) failed to meet criteria (i.e., < 20 reinforcers were received).

### ache^*sb55*/+^ show lower levels of premature responding in long fixed-interval and variable-interval PSIs

The rates of correct responses, omissions and premature responding were comparable with our previously published work with zebrafish (Parker et al., 2012, 2013, 2014). There was a significant main effect of phase for correct responses, *F*_(4, 24)_ = 23.61, *P* < 0.01. *Post-hoc* tests revealed that the proportion of correct responses increased after phase 1 (phase 1 < phases 3, 4 and long-PSI, *P*s < 0.01, but not phase 2, *P* = 0.06) and phase 2 (phase 2 < phases 3, 4 and long-PSI, *P*s > 0.01), but there was no difference between phases 3, 4 and long-PSI (*P*s > 0.14). There was no main effect of strain (ache^*sb55/*+^ = 0.52±0.02, wild-type = 0.52±0.02), *F* < 1, nor a significant phase × strain interaction, *F* < 1.

The rates of premature responding were comparable with our previous studies (Parker et al., 2012, 2013, 2014). There was a significant effect of phase, *F*_(4, 20)_ = 37.17, *P* < 0.01. *Post-hoc* test revealed that phase 1 < phases 2, 3, 4 and long-PSI (*P*s < 0.01), phase 2 < phases 3, 4 and long-PSI (*P*s < 0.01), phase 3 = phase 4 (*P* = 0.3), and subjects performed more premature responses in the long-PSI phase than phases 3 and 4 (*P*s < 0.05). There was also a significant main effect of strain (Figure 2B), *F*_(1, 28)_ = 5.07, *P* = 0.03, with the ache^*sb55/*+^ performing a lower proportion of premature responses than the wild-type. There was no significant phase × strain interaction, *F*_(4, 20)_ = 2.11, *P* = 0.12.

Rates of omissions were again comparable with our previous study (Parker et al., 2012, 2013, 2014). There were significant main effects of phase, *F*_(4, 27)_ = 22.02, *P* < 0.01. *Post-hoc* tests revealed that phase 1 < phases 2, 3, 4 and long-PSI (*P*s < 0.01), and phase 2 > phases 3 and 4 (*P*s < 0.04), but not long-PSI (*P* = 0.3). Phase 3 was not significantly different from phase 4 (*P* = 0.14) but was significantly lower than long-PSI (*P* < 0.03). There was no significant effect of strain (ache^*sb55/*+^ = 0.32±0.02, wild-type = 0.31 ± 0.01), *F* < 1, nor was there a significant phase × strain interaction, *F*_(4, 27)_ = 1.85, *P* = 0.14.

There was a significant effect of phase on the latency to approach the stimulus for correct responses, *F*_(4, 23)_ = 26.91, *P* < 0.01, with subjects taking longer to approach the stimulus in Phase 1 (12.69 ± 0.77 s) than in phases 2 (4.51 ± 0.27 s), 3 (5.31 ± 0.21 s), 4 (5.45 ± 0.19 s) or the long PSI phase (6.0 ± 0.18 s). There was no significant effect of strain, *F* < 1, nor was there a phase × strain interaction, *F*_(4, 23)_ = 1.18, *P* = 0.35.

The number of trials completed in each session during 5-CSRTT training changed significantly according to phase, *F*_(4, 30)_ = 7.96, *P* < 0.01, characterized as fish completing the most trials in phase 3 (40.76 ± 1.23 trials; *P* < 0.01), and fewer trials in the long-PSI phase (31.06 ± 1.29 trials) than in phase 4 (34.89 ± 1.29 trials; *P* < 0.01). There was no main effect of strain, *F* < 1 nor a phase × strain interaction, *F* < 1.

Finally, we carried out a replication with ache^*sb55/*+^ heterozygotes and ache^+∕+^ wild-type siblings. First, fish were trained for 20 sessions (1 s fixed interval PSI), and finally with six, 5 s variable-interval (VI) PSI trials included. ache^*sb55/*+^ showed a significantly lower proportion of premature responses during the VI-PSI trials, *F*_(1, 18)_ = 10.48, *P* = 0.03 (Figure 2C). There were no differences in correct responses (ache^*sb55/*+^ = 0.66±0.03; ache^+∕+^ = 0.61±0.02; *P* = 0.13), nor omissions (ache^*sb55/*+^ = 0.34±0.05; ache^+∕+^ = 0.24±0.03; *P* = 0.1).

### Acute manipulation of cholinergic activity increases performance accuracy but has no effect on anticipatory responding in adult wild-type zebrafish

Figure 2D shows the results of drug administration on 5-CSRTT performance in wild-type fish. There was a significant main effect of drug on correct responses, *F*_(3, 75)_ = 4.01, *P* = 0.01. *Post-hoc* pairwise comparisons (α-adjusted for multiple tests) revealed that there was a significant increase from control in correct responses during the nicotine (*P* = 0.02) but not pilocarpine (*P* = 0.19) or donepezil (*P* = 0.85). There were no differences between nicotine and donepezil (*P* = 0.07), nicotine and pilocarpine (*P* = 0.68) or pilocarpine and donepezil (*P* = 0.53). There were no differences between the drugs' effects in terms of premature response rates (control = 0.126 ± 0.02; nicotine = 0.104 ± 0.03; pilocarpine = 0.103 ± 0.03; donepezil = 0.13 ± 0.03; *F* < 1), nor in terms of omissions (control = 0.08 ± 0.03; nicotine = 0.1 ± 0.04; pilocarpine = 0.1 ± 0.04; donepezil = 0.13 ± 0.04; *F*_(3, 79)_ = 1.22, *P* = 0.3). There were no differences in the total number of trials completed in each session (control = 21.4 ± 0.52; nicotine = 19.2 ± 0.94; pilocarpine = 21.7 ± 0.94; donepezil = 21.4 ± 0.94; *F*_(3, 80)_ = 1.77, *P* = 0.16). Finally, there was no effect of drug on approach latency (control = 8.6 ± 1.3; nicotine = 8.8 ± 1.5; pilocarpine = 9.1 ± 1.5; donepezil = 9.1 ± 1.5; *F* < 1).

### ache^*sb55*∕+^ have down regulation of *chrna2, chrna5*, and *drd2* mRNA at 9 dpf, but no detectable differences in adult expression

Finally, to help understand the mechanisms by which developmental reduction in AChE affected the observed reduction in anticipatory responding, we characterized the gene expression profile of ache^*sb55/*+^ focussing on neural circuits known to be involved in impulse control. Table 3 summarizes the differences in mRNA expression for ache^*sb55/*+^ heterozygotes vs. ache^+∕+^ wild-type siblings. We found that in the ache^*sb55/*+^ heterozygotes, there was robust downregulation in *chrna2, chrna5*, and *drd2* mRNA, the genes that code for the alpha-2, alpha-5 receptor subunits (nAChRa2, nAChRa5), and the dopamine d2 receptor subunit (DRD2), respectively. In the adults, there was no difference in expression of any of the genes we observed.

**Table 3:** **mRNA expression for ache^*sb55*∕+^ vs. *ache*^+∕+^ at 9 dpf and 6 months of age**.

**Gene**	***U***	**N(a)**	**N(b)**	**Uncorrected *P*-value**	**Corrected *P*-value**	**Effect Size (Grissom and Kim, 2012)**	**Direction of change in mRNA expression**
**ADULT (6 MONTHS)**							
*adora2aa*	9	4	5	0.9	1	0.45	–
*chrna2*	17	5	5	0.42	1	0.68	–
*chrna3*	17	5	5	0.42	1	0.68	–
*chrna4*	12	4	5	0.73	1	0.6	–
*chrna5*	18	5	5	0.31	1	0.72	–
*chrna6*	11	4	5	1	1	0.55	–
*chrna7*	14	4	5	0.41	1	0.7	–
*chrnb2*	9.5	4	5	0.9	1	0.475	–
*chrnb3*	8	4	5	0.73	1	0.4	–
*chrnb4*	10	4	5	1	1	0.5	–
*drd1*	9	4	5	0.9	1	0.45	–
*drd2*	11	4	5	1	1	0.55	–
*drd3*	10	5	5	0.69	1	0.4	–
*htrlaa*	12	4	5	0.73	1	0.6	–
*optm1*	13.5	4	5	0.41	1	0.675	–
*slc6a2*	14	4	5	0.41	1	0.7	–
*slc6a3*	14	5	5	0.85	1	0.56	–
*slc6a4*	16	5	5	0.55	1	0.64	–
**9 dpf**							
*adora2aa*	51	8	8	0.05	0.9	0.797	–
***chrna2***	**47**	**8**	**6**	**0.001**	**0.02**	**0.979**	**ache^*sh*55∕+ 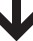 ^**
*chrna3*	33.5	8	8	0.9	1	0.523	–
*chrna4*	46	8	8	0.16	1	0.719	–
***chrnaS***	**94.5**	**8**	**8**	**0.003**	**0.05**	**1.477**	**ache^*sh*55∕+ 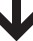 ^**
*chrna6*	50	8	8	0.065	1	0.781	–
*chrna7*	50	8	8	0.065	1	0.781	–
*chrnb2*	52	8	8	0.038	0.68	0.813	–
*chrnb3*	28	8	8	0.72	1	0.438	–
*chrnb4*	50	8	8	0.065	1	0.781	–
*drd1*	54	8	8	0.02	0.36	0.844	–
***drd2***	**53**	**8**	**7**	**0.002**	**0.036**	**0.946**	**ache^*sh*55∕+ 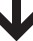 ^**
*drd3*	57	8	8	0.007	0.126	0.891	–
*htrlaa*	54	8	8	0.02	0.36	0.844	–
*optm1*	53	8	8	0.03	0.54	0.828	–
*slc6a2*	55.5	8	8	0.01	0.18	0.867	–
*slc6a3*	45	8	7	0.054	0.972	0.804	–
*slc6a4*	25	8	6	1	1	0.521	–

*Downward arrows represent down-regulation of expression, “–” represents no change. Significant differences indicated by Bold type. All expression ratios are reported relative to bact, rpl13a, and eF1a*.

## Discussion

The aim of this experiment was to test the hypothesis that developmental alterations in cholinergic signaling affect impulse control using a zebrafish model of the commonly used 5-CSRTT with a strain heterozygous for a missense mutation in *ache* (ache^*sb55/*+^). We found that ache^*sb55/*+^ showed a lower proportion of premature responding than ache^*sb55/*+^ siblings and wild-type zebrafish. There were no significant differences in either the number of correct responses, latency to respond, number of trials or the number of omissions, although the ache^*sb55/*+^ appeared to learn faster, collecting more reinforcers during pre-training. Acute reductions of AChE (donepezil) had no significant effects on premature responding, or other 5-CSRTT parameters, and acute administration of a nAChR agonist significantly increased performance accuracy, while having no effect on premature responding. Finally, ache^*sb55/*+^ have a down regulation of *chrna2, chrna5*, and *drd2* mRNA expression at 9 dpf, but no difference in expression in any of the genes we examined in adulthood. Previous studies have shown that high levels of AChE inhibition during development (e.g., with the organophosphate weedkiller chlorpyrifous Middlemore-Risher et al., 2010; Cardona et al., 2011; Oca et al., 2012) increase impulsivity in later life. Collectively, these data suggest the intriguing theory that variation in AChE during development may follow a J-curve with respect to its effects on impulse control, potentially through downstream effects on cholinergic and dopaminergic pathways.

Lesion, neuropsychological, and pharmacological studies have demonstrated that cortical cholinergic projections to mid-brain regions are strongly implicated in sustained attention and in general top-down cognitive control (Sarter et al., 2001). In particular, during 5-CSRTT performance rats display elevated ACh release from the medial pre-frontal cortex (mPFC), and phasic increases in ACh release when a visual distracter was introduced to increase task complexity (Passetti et al., 2000). We did not see any differences in the number of correct responses in our version of the task, but more of the ache^*sb55/*+^ met criteria to move to the 5-CSRTT stage of training, and of those that met criteria, overall performance in terms of reinforcers gained was significantly greater than the wild-type. This finding replicates assessment of this strain's learning previously demonstrated in a T-maze task (Ninkovic et al., 2006). During this initial training stage, despite the strain difference, there was no day × strain interaction, suggesting that ache^*sb55/*+^ learnt at the same rate. It may be that the ache^*sb55/*+^ were more motivated to perform, or habituated faster than the wild-type. This effect was transient, however, disappearing once training started on the 5-CSRTT. We did, however, find evidence for the role of nAChR in task performance, with acute exposure to nicotine (nAChR agonist) increasing the proportion of correct responses in the task. This supports previous data from rodents (Blondel et al., 2000; Hahn and Stolerman, 2002; Young et al., 2004).

A potential mechanism for the observed differences in premature responding may relate to the role of nAChR during early brain development and patterning. nAChR subtypes, in particular α4, α5, α7, β2, and β4, are found early in brain development, and have been suggested to play a role in modulating and mediating early patterning, dendritic outgrowth and synaptogenesis (Hellström-Lindahl et al., 1998). It is possible therefore that reduction in AChE levels, as is characteristic of the ache^*sb55/*+^ heterozygotes, during early brain development alter the distribution of nAChRs thus causing differences in patterning and dendritic morphology. Indeed, in zebrafish, AChE enzymatic activity has been shown to be important for both axon outgrowth and synapse stability, albeit within the neuromuscular projections of the nervous system (Behra et al., 2002; Downes and Granato, 2004).

Chronic reductions of AChE in adult rats with donepezil increases expression of α4 and α7 nAChR (Kume et al., 2005), and ACh-modulated reductions in impulsive action in the 3-CSRTT are mediated by α4 nAChR (Tsutsui-Kimura et al., 2010). Although we did not observe differences either in *chrna4* or *chrna7* here, we did observe robust down regulation of *chrna2* and *chrna5* mRNA expression in the ache^*sb*55∕^+ heterozygotes at 9 dpf, but no differences in adulthood. CHRNA2 and CHRNA5 variants have been shown to predict impulsive responding in response-inhibition in humans (Rigbi et al., 2008), and transgenic mice overexpressing the Chrna3, Chrna5, Chrnb4 gene cluster show a reduction in impulsivity (Viñals et al., 2012). However, the differences in behavior observed in the ache^*sb55/*+^ heterozygotes demonstrate haploinsufficiency of the AChE gene, and thus has implications for the impact of AChE mutations within the human population. Although we are yet to understand the mechanism, this may inform our exploration of potential targets for therapeutics in the future.

The functional properties of nAChRs on catecholaminergic (in particular, dopaminergic) axonal terminals alter during development, highlighting their role in the development of the dopamine system (Azam et al., 2007). It is clear that over-activation of nAChR during early development, e.g., from maternal smoking during pregnancy, can result in an increased risk for impulse control disorders (Button et al., 2007). In addition, as discussed above, excessive inhibition of AChE during development, resulting from exposure to the organophosphate insecticide chlorpyrifos, results in higher impulsivity (Middlemore-Risher et al., 2010; Cardona et al., 2011; Oca et al., 2012). Although this shows a clear link between developmental effects of cholinergic-system disruption and impulsivity, it is not clear at this stage the mechanisms by which subtle alterations, such as are seen with ache^*sb55/*+^, subsequently reduces impulsivity. It is possible that this reflects species-specific differences in patterning during early brain ontogeny, although this seems unlikely based on documented similarities between fish and mammalian cholinergic system development (Xie et al., 2000; Behra et al., 2002).

During development, AChE is transiently involved with aspects of neural patterning and hodological development. For example, during cortical synaptogenesis and development of thalamo-cortical pathways, AChE activity is recorded in various brain regions (Button et al., 2007). The cholinergic system interacts with mid-brain dopamine activity in a number of ways. First, the nucleus accumbens (NAc) is densely innervated by cholinergic projection neurons (Meredith et al., 1989; Woolf, 1991). Second, cholinergic receptors [both muscarinic (mAChR) and nicotinic (nAChR)] are found on ventral tegmental area (VTA) dopamine neurons, suggesting dopaminergic control of cholinergic activity (Clarke and Pert, 1985). Third, mesolimbic cholinergic projection neurons are abundant with dopamine receptors, suggesting cholinergic mediation of dopamine activity (Gronier et al., 2000), creating a feedback loop. Rats characterized as high trait impulsivity based on baseline performance on the 5-CSRTT show a greater tendency for elevated cocaine self-administration (Dalley et al., 2007), increased compulsive cocaine seeking (Belin et al., 2008) and increased relapse to compulsive cocaine seeking following punishment-induced abstinence (Economidou et al., 2009). In addition, high impulsive rats show a reduction in DRD2/DRD3 in the ventral striatum, suggesting a potential biomarker for the addiction phenotype (Dalley et al., 2007). Interestingly, ache^*sb55/*+^ have previously been characterized as showing a decrease in conditioned place preference (CPP) for amphetamine (Ninkovic et al., 2006). It is well-established, through the therapeutic efficacy of dopamine agonists such as methylphenidate in reducing impulsivity in ADHD patients (Barkley, 1997), that impulsivity is, at least in part, related to a reduction in availability of dopamine (Li et al., 2006). It is possible that genetic impairment of AChE in ache^*sb55/*+^ – which results in higher levels of circulating ACh and desensitization of AChRs (Ninkovic et al., 2006) – may act to stabilize dopamine activity (Zhou et al., 2001), thus decreasing impulsive responding. However, although we observed downregulation in *drd2* mRNA in 9 dpf ache^*sb55/*+^ embryos, there was no significant differences in the adults. This requires further exploration in order to elucidate the mechanism.

In rodents, low levels of premature responding in the 5-CSRTT are predictive of animals that show resistance to developing compulsive drug seeking (Belin et al., 2008) and relapse following abstinence (Economidou et al., 2009), and this has been interpreted as these animals showing low levels of trait impulsivity affecting top-down cognitive control (Dalley et al., 2011). The neural circuits of impulsivity are currently not well-understood (Brown et al., 2006; Chang et al., 2012), but these findings suggest that zebrafish, an established genetic model system, offer a means for exploration of this.

Gaining a better understanding of the etiology of psychiatric disease is currently a priority area of research (Campbell, 2010), and with current advances in neuroimaging and huge increases in genetic sequencing power this aim is beginning to be realized. For example, genome-wide association studies (GWAS) are making progress in this regard (Sullivan, 2010), but are limited by uncontrollable factors such as environmental influences and heterogeneity of diseases (Burmeister et al., 2008). Animal models have proved useful in terms of identifying molecular mechanisms of many psychiatric diseases, as symptoms consistent with DSM-IV (APA, 2000) diagnoses of psychiatric disorder have been characterized in many models (Gould and Gottesman, 2006). A better understanding of the molecular mechanisms will be helpful in tailoring treatment options for patients, but also for early identification of at-risk individuals to allow preventative measures to be adopted in the early stages of the disorder (Uhl et al., 2008). Progress in identifying molecular mechanisms, however, has remained slow. This study shows more evidence that zebrafish may be very useful in expediting this process.

In conclusion, this study has found that alterations in *Ache* reduce premature responding in zebrafish on the 5-CSRTT. This effect appears to relate specifically to developmental effects of reduced AChE, as acute exposure to an AChE antagonist had no effect on premature responding in the task. Molecular analyses suggest that the route of action may be through cholinergic interactions with midbrain dopamine systems during development. This study opens the door for potential large-scale forward genetic population screening of mutagenized lines of zebrafish to identify novel alleles for phenotypes such as impulsivity, which is crucial in the search for novel therapeutics and individualized medicine (Jain et al., 2011).

### Conflict of interest statement

The Associate Editor Allan V. Kalueff declares that, despite of having collaborated with the authors Matthew O. Parker and Caroline H. Brennan, the review process was handled objectively. The authors declare that the research was conducted in the absence of any commercial or financial relationships that could be construed as a potential conflict of interest.

## Footnotes

^1^Note that in the rodent version of the 5-CSRTT, there is no gate lifted, and thus the pause prior to the stimulus presentation is an inter-trial interval. In our version of the task, the trial is initiated by the opening of the gate, and we refer to this as pre-stimulus interval (PSI).
